# Alignment among the zygotic cleavage plane, pronuclear axis, and polar axis predicts live birth outcome of blastocyst

**DOI:** 10.1007/s10815-026-03847-9

**Published:** 2026-03-12

**Authors:** Yanhe Liu, Kelli Peirce, Jay Natalwala, Vincent Chapple

**Affiliations:** 1 University of Western Australia, Perth, Australia; 2 Fertility North, Joondalup, Australia

**Keywords:** Zygote, Cleavage plane, Pronuclear, Polar body, Live birth

## Abstract

**Purpose:**

To investigate whether alignment among zygotic cleavage plane (CP), pronuclear (PN) axis, and polar axis (PB) is associated with a higher live birth rate (LBR) in single transferred blastocysts.

**Methods:**

A total of 103 fresh and 138 frozen blastocysts generated via either conventional IVF insemination or intracytoplasmic sperm injection (ICSI) using fresh autologous oocytes between September 2022 and December 2023 were retrospectively analyzed. LBRs were compared according to alignment among three zygotic axes. The PN axis was defined by the longitudinal axis before fading, and the PB axis was determined by the position of the second polar body. Alignment relative to the zygotic CP was classified as CPPN+ or CPPN- for the PN axis, and CPPB+ or CPPB- for the PB axis.

**Results:**

In the fresh transfer dataset, the highest LBR was observed in CPPN+/CPPB+ blastocysts (77.5%), compared with CPPN+/CPPB- (20.0%, *P* < 0.001), CPPN-/CPPB+ (15.2%, *P* < 0.001), and CPPN-/CPPB- (6.7%, *P* < 0.001). Similar patterns were observed in the frozen transfer dataset, where CPPN+/CPPB+ blastocysts again had the highest LBR (48.3%), compared with CPPN+/CPPB- (16.1%, *P* = 0.003), CPPN-/CPPB+ (19.4%, *P* = 0.007), and CPPN-/CPPB2- (11.1%, *P* = 0.005). Logistic regression confirmed CPPN and CPPB alignment as independent predictors of live birth in both datasets, after adjusting for several potential confounders.

**Conclusion:**

Our findings provide preliminary evidence to support zygotic alignment among CP, PN, and PB axes as a potential live birth marker for blastocyst selection.

**Supplementary Information:**

The online version contains supplementary material available at 10.1007/s10815-026-03847-9.

## Introduction

Blastocyst culture is widely used as a tool for embryo selection prior to intrauterine transfer [1]. Recent clinical introduction of time-lapse videography has offered a novel opportunity for further improved embryo selection, with an increasing number of selection algorithms proposed [2–4]. However, a recent well-designed randomized controlled trial did not find significant improvement by either time-lapse culture alone or in combination with the added selection using embryo morphokinetics profiles [5]. Meanwhile, novel viability markers continue to emerge [6, 7], highlighting the growing potential of time-lapse embryo selection via additional newly identified non-invasive time-lapse markers [8].

Recently, there has been a focus on the first mitotic division of the human zygote following its finer morphokinetic profiles being revealed via time-lapse imaging [9, 10]. Among these early morphokinetic features, the alignment among zygotic cleavage plane (CP), pronuclear (PN) axis, and polar (PB) axis was highlighted as potential biomarkers for embryo viability. However, early studies were based on static observations [11], making it a challenge to detect temporal changes in the PN axis orientation, and even impossible to define PB axis being unable to accurately locate the second polar body extrusion. In a 2021 study using a dataset including predominantly day 2/3 transfers, higher pregnancy rates were seen in embryos with aligned zygotic CP and PN axis [12]. A more recent study demonstrated a reduced euploidy rate in blastocysts having misaligned zygotic CP and PN axes [13]. Another study also reported better alignment between zygotic CP and PN in embryos leading to a live birth [14]. However, all the embryos involved in these studies were fertilized via intracytoplasmic sperm injection (ICSI), leaving a knowledge gap in those fertilized via the more natural conventional IVF insemination. Also, live birth data on this topic following blastocyst transfer is also currently scarce in the literature. Furthermore, it remains unclear whether the alignment between zygotic CP and PB axis is indicative of subsequent viability of an embryo. Therefore, using live birth as an endpoint, this study aims to test the hypothesis that alignment among zygotic CP, PN axis, and PB axis is associated with higher live birth rate (LBR) following single blastocyst transfers, regardless of IVF or ICSI insemination.

## Materials and methods

This retrospective study included two separate datasets, namely a fresh transfer dataset including 103 single transferred fresh blastocysts and a frozen transfer dataset comprising 138 single transferred frozen blastocysts. All blastocysts were created between September 2022 and December 2023, using fresh autologous oocytes via either IVF or ICSI. Blastocysts with a morphological grade lower than 3BB, or those involved in double embryo transfers or preimplantation genetic testing, were excluded from the analysis. Time-lapse imaging frequency was set at every 10 min and continuous culture started from either the completion of the 4-h IVF insemination or immediately after ICSI until the end of culture on day 5 or 6. In either the fresh (day 5 only) or frozen (day 5 or 6) dataset, only one blastocyst per patient (first transferred during the forementioned timeframe) was included for analysis to avoid clustering effects at statistical analysis. All clinical and laboratory procedures were conducted according to the standard protocols of the clinic. Table 1 describes baseline characteristics of cycles associated with the included blastocysts in each dataset. Further breakdowns stratified by study group are presented in Supplementary Tables 1 and 2 for the fresh and frozen datasets, respectively. Retrospective data analysis was approved by the Ramsay Health Care Human Research Ethics Committee (2022/ETH/0073).

**Table 1:** Patient characteristics

Parameters	Fresh transfer dataset	Frozen transfer dataset
Number of patients	103	138
Maternal age at oocyte retrieval(years, mean ± SD, min-max)	34.2 ± 3.9 (26–41)	35.3 ± 4.5 (22–46)
Insemination methods		
IVF (%)	45 (43.7%)	55 (40.0%)
ICSI (%)	58 (56.3%)	83 (60.0%)
Number of oocytes collected (mean ± SD, min-max)	9.9 ± 4.4 (1–23)	-
Number of oocytes fertilized (mean ± SD, %)	5.9 ± 3.4 (59.8%, 611/1022)	-
Number of blastocysts formed (%)	334 (54.7%)	-
Number of cells on day 3 (at 66 hpi)		
5 or less (%)	1 (1.0%)	6 (4.3%)
6 (%)	5 (4.9%)	4 (2.9%)
7 (%)	11 (10.7%)	21 (15.2%)
8 (%)	57 (55.3%)	80 (58.0%)
9 or more (%)	29 (28.1%)	27 (19.6%)
Expansion stage at transfer		
Full blastocyst (%)	22 (21.4%)	21 (15.2%)
Expanded (%)	44 (42.7%)	51 (37.0%)
Hatching (%)	37 (35.9%)	65 (47.1%)
Hatched (%)	0	1 (0.7%)
Blastocyst morphology (ICM/TE)		
AA (%)	65 (63.1%)	71 (51.4%)
AB/BA/BB (%)	38 (36.9%)	67 (48.6%)
t2 (hpi, mean ± SD, min-max)	25.4 ± 2.5	25.5 ± 2.8
	(20.1–31.1)	(20.6–37.8)
tB (hpi, mean ± SD, min-max)	101.9 ± 6.6	106.3 ± 10.5
	(84.0–118.6)	(86.4–145.6)
Number of clinical pregnancies (%)	48 (46.6%)	61 (44.2%)
Number of miscarriages (%)	8 (16.7%)	20 (32.8%)
Number of live births (%)	40 (38.8%)	41 (29.7%)

*hpi* hours post insemination, *ICM* inner cell mass, *TE* trophectoderm, *tB* timing of blastulation, *min* minimal value, *max* maximal value

### Gamete preparation, insemination, and embryo culture

Ovarian stimulation, transvaginal oocyte aspiration, and sperm preparation were performed according to previous publication [15]. In particular, sperm samples were processed using either the swim-up or density gradient centrifugation protocols, depending on the initial microscopic assessment. To address the artificial nature of sperm entry in ICSI, we extended the investigation to conventional IVF for comparison. For IVF insemination, cumulus-oocyte complexes (COCs) were co-incubated with the prepared sperm sample for 4 h in G-IVF™ Plus media (Vitrolife, Sweden) overlaid with Ovoil™ (Vitrolife) at 6% CO_2_, 5% O_2_, and 89% N_2_ at 37℃. Oocytes were then separated from cumulus cells and sperm, and placed into G-TL™ Plus media (Vitrolife) for uninterrupted culture up to day 6 at 37℃ under 6% CO_2_, 5% O_2_, and 89% N_2_ in the Embryoscope+ incubator (Vitrolife), a non-humidified system. COCs for ICSI insemination were denuded by brief exposure to Synvitro Hyadase (Origio, Denmark) for a maximum of 10 s, followed by mechanical removal of cumulus cells from the oocytes. Metaphase II oocytes were injected with a single spermatozoon, with the first polar body oriented at either 12- or 6-o’clock, before uninterrupted culture in the Embryoscope+ incubator up to day 6. Fertilization was confirmed 16–20 h post insemination by reviewing the overnight time-lapse footage. Only oocytes exhibiting two pronuclei and two polar bodies were classified as normal fertilized.

### Fresh blastocyst transfer, vitrification/warming of blastocysts, and frozen transfer

Blastocysts were assessed according to the Gardner system [1] on day 5 or day 6. Those exhibiting at least stage-3 expansion and A- or B-grade inner cell mass and trophectoderm were prioritized for transfer or cryopreservation. The embryo transfer procedure was conducted as previously described [15]. Suitable blastocysts were vitrified using the Rapid-i™ device and RapidVit™ Blast media (Vitrolife) and subsequently warmed using the RapidWarm™ Blast media (Vitrolife), in accordance with the manufacturer’s protocol. All frozen blastocysts were transferred 2 to 4 h post warm as previously described [16]. All pregnancies were followed up until birth with a definitive outcome recorded for each blastocyst. Biochemical pregnancy was defined as a β-hCG level > 25 mIU/ml 14 days after ovulation. Clinical pregnancy was confirmed by the presence of a gestational sac on ultrasound. Miscarriage was defined as pregnancy loss before 20 weeks of gestation.

### Time-lapse annotation for zygotic alignment

**Fig. 1:**
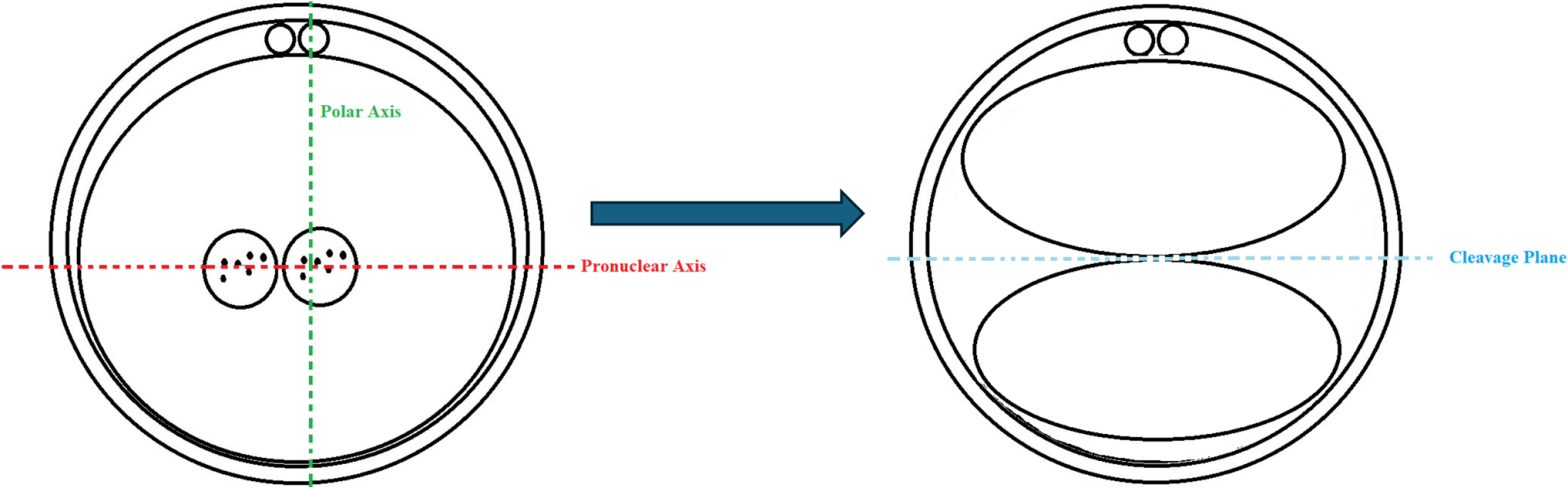
Cleavage plane, pronuclear axis, and polar axis

**Fig. 2:**
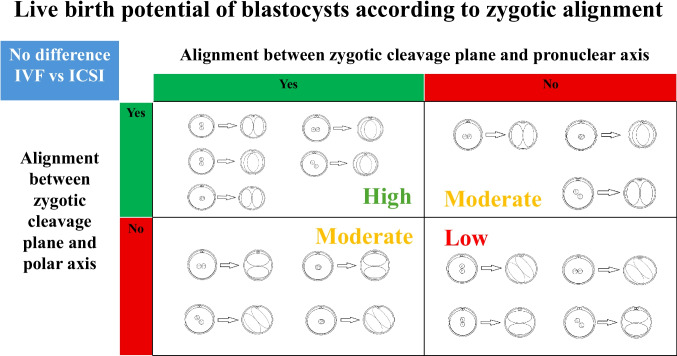
Classification of blastocysts according to zygotic alignment outcomes with possible phenotypes illustrated for each category

All annotations for zygotic alignment were retrospectively carried out by the same embryologist (YL), who was blinded to birth outcomes and blastocyst grades at data collection. The PN axis was defined by their longitudinal axis before fading (last image before pronuclear breakdown), while the PB axis was represented by either (a) the position of the second polar body extrusion or (b) where the female PN emerged if the extrusion footage was unavailable due to IVF insemination (Fig. 1, Video 1). Alignment relative to the zygotic CP was classified as CPPN+ (aligned cleavage plane and pronuclear axis) or CPPN- (misaligned cleavage plane and pronuclear axis) for the PN axis, and CPPB+ (aligned cleavage plane and polar axis) or CPPB- (misaligned cleavage plane and polar axis) for the PB axis. A total of four categories were classified by combining alignment outcomes of CPPN and CPPB, comprising the CPPN+/CPPB+, CPPN+/CPPB-, CPPN-/CPPB+, and CPPN-/CPPB- subgroups. Examples under each category are illustrated in Fig. 2, including zygotes in which the two pronuclei appeared to overlap from the viewing angle, reflecting their three-dimensional nature. In cases where the polar bodies were fragmented and the location of the second polar body could not be confidently identified, the site at which the maternal pronucleus initially emerged was used to determine its position. All zygotes were successfully annotated using the 11 focal planes captured by the Embryoscope+ system; therefore, no embryos were excluded due to focal-plane limitations. An example of time-lapse footage demonstrating CPPN+/CPPB+ is also presented in Video 2, captured from an embryo leading to a live birth.

### Statistical analysis

Qualitative variables, including LBRs, were expressed as proportions and compared between groups using Fisher’s exact test. Continuous parameters were assessed via Student’s *T*-test. Multivariate logistic regression was employed to evaluate independent associations between studied factors and the subsequent live birth and miscarriage outcomes, adjusting for maternal age at oocyte retrieval, insemination methods, day 3 cell count, blastocyst expansion degree, morphology of ICM and TE, and timings of the two-cell stage and blastulation. Statistics were expressed via adjusted odds ratios (aOR) and 95% confidence interval (CI). Statistical analysis was conducted using Statistical Package for the Social Sciences (version 25, IBM, Washington, USA) with *P* < 0.05 considered as statistically significant.

## Results

A total of 81 (33.6%) live births were recorded from the 241 single blastocyst transfer cycles (Table 1), including 40 (38.8%) from the fresh transfer subset (*n* = 103, aged 34.2 ± 3.9 years) and 41 (29.7%) from the frozen transfer subset (*n* = 138, aged 35.3 ± 4.5 years).

### Fresh transfer dataset

In the fresh transfer dataset (Table 2), the highest LBR was observed in CPPN+/CPPB+ blastocysts (77.5%), compared to CPPN+/CPPB- (20.0%, *P* < 0.001), CPPN-/CPPB+ (15.2%, *P* < 0.001), and CPPN-/CPPB- (6.7%, *P* < 0.001). Maternal ages did not differ significantly between groups (*P* > 0.05, respectively).

**Table 2:** Live birth rates and maternal ages (years) according to alignment among zygotic cleavage plane, pronuclear axis, and polar axis in freshly transferred blastocysts (*n* = 103)

		Live birth rates and maternal ages according to alignment outcomes between zygotic cleavage plane and pronuclear axis (CPPN)		
		Yes (CPPN+)	No (CPPN-)	Total
Live birth rates and maternal ages according to alignment outcomes between zygotic cleavage plane and polar axis (CPPB)	Yes (CPPB+)	77.5% (31/40) 33.8 ± 4.3	15.2% (5/33)^ 34.8 ± 4.0	49.3% (36/73) 34.3 ± 4.2
	No (CPPB-)	20.0% (3/15)^ 33.8 ± 3.4	6.7% (1/15)^ 34.6 ± 2.9	13.3% (4/30) 34.2 ± 3.1
	Total	61.8% (34/55) 33.8 ± 4.1	12.5% (6/48) 34.7 ± 3.7	38.8% (40/103) 34.2 ± 3.9

Chi-squared analysis was used to test statistical significance between different groups. ^*P* < 0.001 compared with CPPN+CPPB+. No statistically significant differences (*P* > 0.05) were detected via Student’s *T*-test in maternal age comparisons between groups

### Frozen transfer dataset

In the frozen transfer dataset (Table 3), the highest LBR was again observed in CPPN+/CPPB+ blastocysts (48.3%), compared to CPPN+/CPPB- (16.1%, *P* = 0.003), CPPN-/CPPB+ (19.4%, *P* = 0.007), and CPPN-/CPPB2- (11.1%, *P* = 0.005). However, maternal age was significantly older in the CPPN-CPPB+ group compared with the CPPN+CPPB+ group (*P* = 0.039).

**Table 3:** Live birth rates and maternal ages (years) according to alignment among zygotic cleavage plane, pronuclear axis, and polar axis in frozen transferred blastocysts (*n* = 138)

		Live birth rates and maternal ages according to alignment outcomes between zygotic cleavage plane and pronuclear axis (CPPN)		
		Yes (CPPN+)	No (CPPN-)	Total
Live birth rates and maternal ages according to alignment outcomes between zygotic cleavage plane and polar axis (CPPB)	Yes (CPPB+)	48.3% (28/58) 34.5 ± 4.1	19.4% (6/31)^ 36.5 ± 4.6^^	38.2% (34/89) 35.2 ± 4.4
	No (CPPB-)	16.1% (5/31)^ 35.0 ± 4.8	11.1% (2/18)^ 36.5 ± 4.4	14.3% (7/49) 35.6 ± 4.7
	Total	37.1% (33/89) 34.7 ± 4.3	16.3% (8/49) 36.5 ± 4.5	28.7% (41/138) 35.3 ± 4.5

Chi-squared analysis was used to test statistical significance in live birth rates between different groups. ^*P* < 0.01 compared with CPPN+CPPB+. Student’s *T*-test was used to test statistical significance in maternal age at oocyte retrieval between different groups. ^^*P* < 0.05 compared with CPPN+CPPB+

### Insemination methods

Table 4 shows a detailed breakdown in LBRs of each subgroup according to zygotic alignment, comparing the IVF- and ICSI-originated blastocysts in either the fresh or frozen transfer dataset. No significant difference (*P* > 0.05) was detected in LBRs between the IVF and ICSI groups.

**Table 4:** Comparison of live birth rates between insemination methods (IVF vs ICSI) in either fresh or frozen blastocyst transfers according to different alignment outcomes among zygotic cleavage plane, pronuclear axis, and polar axis

		IVF	ICSI	Total
Fresh blastocysts (*n* = 103)	CPPN+ CPPN-	64.3% (18/28) 5.9% (1/17)	59.3% (16/27) 16.1% (5/31)	61.8% (34/55) 12.5% (6/48)
	CPPB+ CPPB-	50.0% (17/34) 18.2% (2/11)	48.7% (19/39) 10.5% (2/19)	49.3% (36/73) 13.3% (4/30)
Frozen blastocysts (*n* = 138)	CPPN+ CPPN-	42.1% (16/38) 17.6% (3/17)	33.3% (17/51) 15.6% (5/32)	37.1% (33/89) 16.3% (8/49)
	CPPB+ CPPB-	47.1% (16/34) 14.3% (3/21)	32.7% (18/55) 14.3% (4/28)	38.2% (34/89) 14.3% (7/49)

Chi-squared analysis showed no significant difference (*p* > 0.05) between IVF and ICSI groups in any of the above subgroups. *CPPN+,* aligned cleavage plane and pronuclear axis; *CPPN-*, misaligned cleavage plane and pronuclear axis; *CPPB+*, aligned cleavage plane and polar axis; *CPPB-*, misaligned cleavage plane and polar axis

### Logistic regression

In the fresh transfer dataset, both CPPN (aOR = 17.543, 95% CI 4.661–66.024, *P* < 0.001) and CPPB (aOR = 13.514, 95% CI 3.080–59.305, *P* < 0.001) alignments were independently associated with higher LBRs after adjusting for potential confounding factors, including maternal age. Similarly, in the frozen transfer dataset, independent associations were identified for CPPN (aOR = 2.926, 95% CI 1.097–7.801, *P* = 0.032) and CPPB (aOR = 3.752, 95% CI 1.416–9.940, *P* = 0.008). However, despite trends observed in Supplementary Tables 1 and 2, no statistically significant association (*P* > 0.05) was detected between zygotic alignment and subsequent miscarriage risk among clinical pregnancies in either dataset, except for CPPN in the frozen transfer dataset (aOR = 0.233, 95% CI 0.055–0.980, *P* = 0.047).

## Discussion

Our data support the hypothesis that alignment among zygotic CP, PN axis, and PB axis is associated with higher LBRs following single blastocyst transfers. This is in agreement with previous studies [12, 13, 17], which reported that alignment between the CP and PN axis correlates with increased euploidy or pregnancy rates. While a mouse study suggested a potential role of the sperm entry site in determining the zygotic CP [18], human studies to date have been limited to embryos derived exclusively from ICSI [12, 13, 17]. A recent primate study demonstrated a difference in the cleavage plane between zygotes generated via conventional IVF and those generated via ICSI [19]. However, our findings demonstrate that the correlation between zygotic alignment and live birth is independent of the insemination method, thereby bridging an important gap in the current literature. This is, to some extent, consistent with an earlier study based on static morphology, which reported that pronuclear orientation relative to the polar body position was similar in human zygotes derived from both conventional IVF and ICSI [20].

Moreover, our study included an unselected patient cohort, enhancing the generalizability of our findings compared to studies involving selected patient populations, such as those limited to PGT-eligible patients [13]. A further strength of our study is the use of live birth as the primary outcome, which is widely regarded as the most clinically relevant endpoint in IVF research, surpassing surrogate markers such as euploidy or pregnancy rates [12, 13]. Our analysis also accounted for potential confounding variables, including insemination methods, maternal age at oocyte retrieval, day 3 cell count, and blastocyst morphology. In particular, maternal age is known to be associated with reduced biological functionality of the oocyte, potentially impacting subsequent first cytokinesis and zygotic alignment. These adjustments reinforce the robustness of zygotic alignment as a potential complementary selection marker to conventional morphology assessment [21]. The degree of fragmentation on day 3 was not included as a confounder due to insufficient heterogeneity, as the blastocysts in our datasets generally exhibited low degree of fragmentation and were consistently ranked among the top embryos within each cohort.

The biological significance of zygotic alignment between the CP and PN axis remains poorly understood. Using immunofluorescence, it was identified that two pericentric signals were located at the interface between the paternal and maternal pronuclei in the majority of analyzed zygotes [12]. This finding underscores the potential importance of spatial alignment between the PN axis and the first mitotic spindle, which is established by the corresponding centrosome at syngamy. Accurate chromosomal segregation during the first zygotic cleavage is paramount [22] and segregation errors may lead to aneuploid daughter cells [23]. Therefore, it is speculated that alignment between the zygotic CP and PN axis could promote correct zygotic cleavage. In our study, no association was observed between maternal age and either CPPN or CPPB alignment, consistent with the findings of a previous study [13]. This further implies that mitotic-origin aneuploidy may be independent of maternal age, in contrast to the well-established age-related aneuploidy risk associated with meiotic errors.

Zygotic polarity is well-established in animal models, with the second PB marking the embryonic pole, opposite the abembryonic pole [24], although it remains unclear in humans. It is hypothesized that misalignment between the zygotic CP and PB axis may lead to an unequal distribution of polarized cytoplasmic determinants between daughter cells, potentially impairing subsequent embryonic development [25]. However, clinical studies testing this hypothesis remain limited. A pilot study based on static observations examined the relationship between the angle of the two polar bodies at the zygotic stage and subsequent developmental potential [26], without directly assessing the cleavage plane. Moreover, as acknowledged by the authors, reliance on static images prevented the evaluation of the temporal dynamics of polar body movements. In contrast, time-lapse technology utilized in our study overcame these limitations, enabling reliable identification of polar body positions and clearer differentiation between the first and second polar bodies. A recent conference abstract suggested that the zygotic CP is independent of the PB axis, although this statement was not supported by a direct assessment of CPPB alignment [14]. Instead, their analysis was limited to evaluating the angle between the PN and PB axes. In contrast, our study employed the zygotic CP as a direct reference for its alignment with the PB axis, providing a more robust evaluation of this relationship. However, future studies taking into account the size of the resulting blastomeres following the first cleavage could provide additional insights into the cytoplasmic polarization hypothesis.

Our study outlined detailed protocols for defining zygotic CP, PN axis, and PB axis, offering a potential framework for standardization and improved comparability in future research. Recognizing the three-dimensional architecture of human zygotes, we presented all possible alignment patterns from various viewing angles in Fig. 2, categorized according to different alignment outcomes. We also established a practical protocol to define the PB axis in zygotes fertilized via conventional IVF, using the emergence location of maternal PN as a marker to distinguish the second PB from the first (Video 1). Furthermore, given the highly dynamic nature of PN axis orientation prior to PN breakdown, it is critical to define the PN axis based solely on its final orientation immediately before fading (Video 1).

Although both our fresh and frozen transfer datasets demonstrated similar trends, we observed a relatively lower LBR for the CPPN+/CPPB+ category within the frozen transfer dataset (48.4%) compared to the fresh transfer dataset (77.5%). This discrepancy may be partially attributed to several factors: (a) blastocysts in the frozen transfer dataset were generally graded with lower preference than those selected for fresh transfer (as shown in Table 1); (b) the frozen transfer dataset included a higher proportion of slower-developing blastocysts (i.e., day 6); and (c) potential effects associated with vitrification and warming procedures. Nevertheless, the associations observed in our study remained statistically relevant across both fresh and frozen transfers, reinforcing the potential value of zygotic alignment, specifically among the CP, PN axis, and PB axis, as a novel marker in future interpretable blastocyst selection models [27].

Several limitations of our study should be acknowledged. First, although the sample size provided sufficient statistical power to detect differences in the primary comparisons, it is still considered relatively small with potentially increased type I and type II errors. Second, the retrospective design introduces the possibility of unmeasured or residual confounding variables beyond those adjusted for in our regression analysis. Additionally, the annotation of zygotic CP, PN axis, and PB axis involves a degree of subjectivity. To minimize variability, all annotations in this study were performed by a single operator, thereby eliminating inter-operator variation. However, broader clinical implementation of this selection marker will require formal assessment of inter-operator reproducibility, supported by a structured training program to ensure consistent annotation. In the long term, automated annotation assisted by artificial intelligence technology offers a promising solution, enhancing both objectivity and efficiency.

In conclusion, in this exploratory pilot study, we observed that alignment of the CP, PN, and PB axes in the zygote is associated with higher live birth potential after blastocyst transfer. Although the sample size is relatively limited, these preliminary yet demonstrative results indicate that axis alignment may serve as a valuable complementary biomarker to improve blastocyst selection strategies. Furthermore, our data help bridge an important gap in the literature by demonstrating that IVF-derived zygotes exhibit alignment patterns comparable to their ICSI-derived counterparts.

## Supplementary Information

Below is the link to the electronic supplementary material.

ESM1 (MP4 3.13 MB)

ESM2 (MP4 541 KB)

ESM3 (DOCX 19.8 KB)

ESM4 (DOCX 20.3 KB)

## Data Availability

Data would be made available upon reasonable requests to the corresponding author.
