# Supplementary material for: Alignment among the zygotic cleavage plane, pronuclear axis, and polar axis predicts live birth outcome of blastocyst
Source: J Assist Reprod Genet. 2026 Mar 12;43(5):1573–80. doi: 10.1007/s10815-026-03847-9 (PMC13221487; doi:10.1007/s10815-026-03847-9)
Supplement: Supplementary file 3 — (DOCX 19.8 KB) [file 10815_2026_3847_MOESM3_ESM.docx]

**Supplementary Table 1. Patient characteristics of fresh cycles**

| **Parameters** | **Study groups for the fresh transfer dataset** | | | | **Total** |
| --- | --- | --- | --- | --- | --- |
|  | **CPPN+/CPPB+** | **CPPN+/CPPB-** | **CPPN-/CPPB+** | **CPPN-/CPPB-** |  |
| Number of patients  Maternal age at oocyte retrieval  (years, mean±SD, min-max)  Insemination methods  IVF (%)  ICSI (%)  Number of oocytes collected (mean±SD, min-max)  Number of oocytes fertilized (mean±SD, %)  Number of cells on Day 3 (%)  5 or less  6  7  8  9 or more  Expansion stage at transfer (%)  Full blastocyst  Expanded  Hatching  Hatched  Blastocyst morphology (ICM/TE)  AA (%)  AB/BA/BB (%)  t2 (hpi, mean±SD, min-max)  tB (hpi, mean±SD, min-max)  Number of clinical pregnancies (%)  Number of miscarriages (%)  Number of live births (%) | 40  33.8±4.3 (26-41)  22 (55.0%)  18 (45.0%)  10.3±4.8 (3-23)  5.6±3.1  (54.9%, 225/410)  0  2 (5.0%)  3 (7.5%)  20 (50.0%)  15 (37.5%)  5 (12.5%)  18 (45.0%)  17 (42.5%)  0  28 (70.0%)  12 (30.0%)  25.2±2.4  (20.9-31.1)  101.1±6.6  (84.0-118.6)  33 (82.5%)  2 (6.1%)  31 (77.5%) | 15  33.8±3.4 (27-39)  6 (40.0%)  9 (60.0%)  10.1±4.5 (4-21)  5.8±3.8  (57.4%, 87/152)  0  0  2 (13.3%)  10 (66.7%)  3 (20.0%)  3 (20.0%)  8 (53.3%)  4 (26.7%)  0  10 (66.7%)  5 (33.3%)  25.3±2.6  (20.1-30.4)  101.0±7.6  (89.4-116.0)  3 (20.0%)  0  3 (20.0%) | 33  34.8±4.0 (26-41)  12 (36.4%)  21 (63.6)  9.8±4.1 (1-19)  6.2±3.5  (63.0%, 204/324)  1 (3.0%)  3 (9.1%)  4 (12.1%)  17 (51.5%)  8 (24.3%)  9 (27.3%)  14 (42.4%)  10 (30.3%)  0  20 (60.6%)  13 (39.4%)  25.6±2.7  (21.0-31.1)  102.8±6.5  (92.3-116.7)  10 (30.3%)  5 (50.0%)  5 (15.2%) | 15  34.6±2.9 (28-39)  5 (33.3%)  10 (66.7%)  9.1±4.0 (3-18)  6.3±3.7  (69.9%, 95/136)  0  0  2 (13.3%)  10 (66.7%)  3 (20.0%)  5 (33.3%)  4 (26.7%)  6 (40.0%)  0  7 (46.7%)  8 (53.3%)  25.2±2.0  (21.4-28.4)  103.2±5.7  (94.1-115.0)  2 (13.3%)  1 (50.0%)  1 (6.7%) | 103  34.2±3.9 (26-41)  45 (43.7%)  58 (56.3%)  9.9±4.4 (1-23)  5.9±3.4  (59.8%, 611/1022)  1 (1.0%)  5 (4.9%)  11 (10.7%)  57 (55.3%)  29 (28.1%)  22 (21.4%)  44 (42.7%)  37 (35.9%)  0  65 (63.1%)  38 (36.9%)  25.4±2.5  (20.1-31.1)  101.9±6.6  (84.0-118.6)  48 (46.6%)  8 (16.7%)  40 (38.8%) |

Note: hpi=hours post insemination, ICM=inner cell mass, TE=trophectoderm, t2=timing of 2-cell stage, tB=timing of blastulation, min=minimal value, max=maximal value, CPPN+, aligned cleavage plane and pronuclear axis; CPPN-, misaligned cleavage plane and pronuclear axis; CPPB+, aligned cleavage plane and polar axis; CPPB-, misaligned cleavage plane and polar axis.
