# Supplementary material for: Alignment among the zygotic cleavage plane, pronuclear axis, and polar axis predicts live birth outcome of blastocyst
Source: J Assist Reprod Genet. 2026 Mar 12;43(5):1573–80. doi: 10.1007/s10815-026-03847-9 (PMC13221487; doi:10.1007/s10815-026-03847-9)
Supplement: Supplementary file 4 — (DOCX 20.3 KB) [file 10815_2026_3847_MOESM4_ESM.docx]

**Supplementary Table 2. Patient characteristics of frozen cycles**

| **Parameters** | **Study groups for the frozen transfer dataset** | | | | **Total** |
| --- | --- | --- | --- | --- | --- |
|  | **CPPN+/CPPB+** | **CPPN+/CPPB-** | **CPPN-/CPPB+** | **CPPN-/CPPB-** |  |
| Number of patients  Maternal age at oocyte retrieval  (years, mean±SD, min-max)  Insemination methods  IVF (%)  ICSI (%)  Number of cells on Day 3 (%)  5 or less  6  7  8  9 or more  Expansion stage at transfer (%)  Full blastocyst  Expanded  Hatching  Hatched  Blastocyst morphology (ICM/TE)  AA (%)  AB/BA/BB (%)  t2 (hpi, mean±SD, min-max)  tB (hpi, mean±SD, min-max)  Number of clinical pregnancies (%)  Number of miscarriages (%)  Number of live births (%) | 58  34.5±4.1  (22-42)  27 (46.6%)  31 (53.4%)  1 (1.7%)  3 (5.2%)  6 (10.3%)  31 (53.5%)  17 (29.3%)  3 (5.2%)  26 (44.8%)  28 (48.3%)  1 (1.7%)  30 (51.7%)  28 (48.3%)  25.4±2.5  (20.6-31.9)  104.2±8.8  (86.4-124.2)  36 (62.1%)  8 (22.2%)  28 (48.3%) | 31  35.0±4.8  (26-42)  11 (35.5%)  20 (64.5%)  2 (6.4%)  0  6 (19.4%)  19 (61.3%)  4 (12.9%)  6 (19.4%)  10 (32.3%)  15 (48.3%)  0  16 (51.6%)  15 (48.4%)  25.3±2.2  (21.6-29.9)  110.1±13.3  (88.0-145.6)  8 (25.8%)  3 (37.5%)  5 (16.1%) | 31  36.5±4.6  (28-46)  7 (22.6%)  24 (77.4%)  2 (6.4%)  0  8 (25.8%)  18 (58.1%)  3 (9.7%)  8 (25.8%)  10 (32.3%)  13 (41.9%)  0  17 (54.8%)  14 (45.2%)  25.5±2.8  (21.1-31.7)  106.7±8.6  (89.6-127.8)  12 (38.7%)  6 (50.0%)  6 (19.4%) | 18  36.5±4.4  (26-44)  10 (55.6%)  8 (44.4%)  1 (5.6%)  1 (5.6%)  1 (5.6%)  12 (66.6%)  3 (16.6%)  4 (22.2%)  5 (27.8%)  9 (50.0%)  0  8 (44.4%)  10 (55.6%)  26.2±4.0  (21.9-37.8)  105.7±12.3  (92.0-136.1)  5 (27.8%)  3 (60.0%)  2 (11.1%) | 138  35.3±4.5  (22-46)  55 (40.0%)  83 (60.0%)  6 (4.3%)  4 (2.9%)  21 (15.2%)  80 (58.0%)  27 (19.6%)  21 (15.2%)  51 (37.0%)  65 (47.1%)  1 (0.7%)  71 (51.4%)  67 (48.6%)  25.5±2.8  (20.6-37.8)  106.3±10.5  (86.4-145.6)  61 (44.2%)  20 (32.8%)  41 (29.7%) |

Note: hpi=hours post insemination, ICM=inner cell mass, TE=trophectoderm, t2=timing of 2-cell stage, tB=timing of blastulation, min=minimal value, max=maximal value, CPPN+, aligned cleavage plane and pronuclear axis; CPPN-, misaligned cleavage plane and pronuclear axis; CPPB+, aligned cleavage plane and polar axis; CPPB-, misaligned cleavage plane and polar axis.
